# Exploring the Use of a Length AI Algorithm to Estimate Children’s Length from Smartphone Images in a Real-World Setting: Algorithm Development and Usability Study

**DOI:** 10.2196/59564

**Published:** 2024-11-22

**Authors:** Mei Chien Chua, Matthew Hadimaja, Jill Wong, Sankha Subhra Mukherjee, Agathe Foussat, Daniel Chan, Umesh Nandal, Fabian Yap

**Affiliations:** 1 Department of Neonatology KK Women's and Children's Hospital Singapore Singapore; 2 Duke-NUS Medical School Singapore Singapore; 3 Lee Kong Chian School of Medicine Nanyang Technological University Singapore Singapore; 4 Yong Loo Lin School of Medicine National University of Singapore Singapore Singapore; 5 Danone Nutricia Research Singapore Singapore; 6 Endocrinology Service, Division of Medicine KK Women's and Children's Hospital Singapore Singapore; 7 Danone Nutricia Research Utrecht Netherlands

**Keywords:** computer vision, length estimation, artificial intelligence, smartphone images, children, AI, algorithm, imaging, height, length, measure, pediatric, infant, neonatal, newborn, smartphone, mHealth, mobile health, mobile phone

## Abstract

**Background:**

Length measurement in young children younger than 18 months is important for monitoring growth and development. Accurate length measurement requires proper equipment, standardized methods, and trained personnel. In addition, length measurement requires young children’s cooperation, making it particularly challenging during infancy and toddlerhood.

**Objective:**

This study aimed to develop a length artificial intelligence (LAI) algorithm to aid users in determining recumbent length conveniently from smartphone images and explore its performance and suitability for personal and clinical use.

**Methods:**

This proof-of-concept study in healthy children (aged 0-18 months) was performed at KK Women’s and Children’s Hospital, Singapore, from November 2021 to March 2022. Smartphone images were taken by parents and investigators. Standardized length-board measurements were taken by trained investigators. Performance was evaluated by comparing the tool’s image-based length estimations with length-board measurements (bias [mean error, mean difference between measured and predicted length]; absolute error [magnitude of error]). Prediction performance was evaluated on an individual-image basis and participant-averaged basis. User experience was collected through questionnaires.

**Results:**

A total of 215 participants (median age 4.4, IQR 1.9-9.7 months) were included. The tool produced a length prediction for 99.4% (2211/2224) of photos analyzed. The mean absolute error was 2.47 cm for individual image predictions and 1.77 cm for participant-averaged predictions. Investigators and parents reported no difficulties in capturing the required photos for most participants (182/215, 84.7% participants and 144/200, 72% participants, respectively).

**Conclusions:**

The LAI algorithm is an accessible and novel way of estimating children’s length from smartphone images without the need for specialized equipment or trained personnel. The LAI algorithm’s current performance and ease of use suggest its potential for use by parents or caregivers with an accuracy approaching what is typically achieved in general clinics or community health settings. The results show that the algorithm is acceptable for use in a personal setting, serving as a proof of concept for use in clinical settings.

**Trial Registration:**

ClinicalTrials.gov NCT05079776; https://clinicaltrials.gov/ct2/show/NCT05079776

## Introduction

Regular and accurate measurement of anthropometric parameters in young children is important for monitoring growth and development, and for facilitating timely interventions to ensure appropriate growth [1,2]. Body length measurements are required for two key World Health Organization (WHO) growth standards: (1) length for age and (2) weight for length [3]. Accurate length measurement requires specialized equipment (a properly calibrated length board), skilled personnel, and a cooperative child [4-7]. Studies have reported inaccuracy and variability of length measurements, even in clinical settings [5,6,8,9]. In practice, achieving the “gold-standard” level of accuracy with the standard method is very challenging for untrained or inexperienced personnel. Although parents and caregivers want to track their children’s growth closely, many find measuring their child’s length at home technically challenging. Thus, there is an unmet need to develop a tool that is easy to use and addresses the key obstacles in taking accurate length measurements. This can potentially be upscaled and deployed in both personal or home and clinical environments.

Mobile devices are increasingly used for fast and efficient collection of real-world data, especially through smartphone images. Advances in artificial intelligence and computer vision technology, particularly deep learning, have enabled complex image recognition and prediction tasks to be performed on such image data [10-14]. These include challenging tasks such as predicting the physical size of a 3D object from one or more 2D images, which could translate to clinical use in determining a person’s anthropometric measurements. Most of the existing image-based approaches focus on predicting the standing height of adults [15,16], although a couple of approaches for predicting the recumbent length of young children have been proposed [17,18]. Unlike manual measurements, these automated image-based approaches do not rely on standardized positioning of the child’s body but must overcome certain challenges to produce accurate predictions. The relevant body parts must be automatically identified within the image by locating key landmarks such as the head and limb joints. The length prediction method must also account for body parts having variable orientation and distance from the camera, which affects their apparent lengths in the image. Image artifacts, such as blurring due to the child’s movement, must also be detected and accounted for. One group proposed a stereoscopic vision system that uses 2 cameras to photograph the child simultaneously from different angles and estimates the child’s body length based on the 2 images using the parallax principle [17]. Another group proposed a method involving the detection of customized round markers placed on the child’s body before image capture. The markers allow both the detection of body landmarks in the image and the estimation of their 3D position relative to the camera; these are used to predict overall body length [18]. However, neither approach fully overcomes all current challenges with child length measurement since they still require specialized equipment or additional manual setup.

We developed a length artificial intelligence (LAI) algorithm to automatically predict children’s length from smartphone images. To our knowledge, the LAI algorithm is the first approach that does not require specialized equipment or precise placement of body segment markers for length prediction. This innovative approach could make it much more practical and convenient for parents or caregivers to take regular length measurements for their children. In this proof-of-concept study, we examined the LAI algorithm’s performance for automated length prediction and compared its performance with international standards such as those from the WHO [19-21] and measurements taken in general or community health clinic settings [8,22]. These comparisons allowed us to assess the feasibility of using the LAI algorithm in scenarios where specialized equipment and skilled personnel are unavailable. In addition, we explored users’ experience and expectations for a digital measurement tool that could be used in home or clinic environments.

## Methods

### Study Design and Participants

An exploratory, observational, cross-sectional pilot study was conducted between November 2021 and March 2022 at KK Women’s and Children’s Hospital, Singapore. The study was prospectively registered at ClinicalTrials.gov (NCT05079776).

Eligible participants were children aged between 0 and 18 months whose parents (1) had a smartphone or tablet with access to the internet, (2) were able to complete the study questionnaires, and (3) took and uploaded images onto an online form. Children who were unable to undergo length measurement by the standardized technique recommended by the WHO [23] (eg, children with structural abnormalities of the lower limbs or orthopedic conditions such as club foot and hip dysplasia) were excluded from the study.

The study duration was a maximum of 2 days. On day 1 (clinic setting), investigators measured the participant’s body length using the standardized WHO length measurement technique [23]. They then used a smartphone to take 6 top-view photos of the participant in a supine position. Each photo included a standard-size reference card. On day 1 or 2 (home setting), parents took and uploaded 6 smartphone photos of the participant in a supine position with the reference card. Investigators and parents were given a list of image quality requirements and guidelines for capturing good-quality images. Parents and investigators completed their respective user experience questionnaires after the image upload process.

### Ethical Considerations

KK Women’s and Children’s Hospital’s independent ethics committee approved the study before its initiation (approval no.: 2021/2540). The study was conducted in accordance with good clinical practice, the Declaration of Helsinki, and the local laws and regulations of Singapore. Written informed consent was obtained from the parent(s) of each participant before any study-related activities were undertaken. The participants’ parent(s) received vouchers (equivalent to 30 Singapore dollars, or US $22.32) as a token of appreciation for participating in the study. Participant data were pseudonymized for analysis.

### Study Assessments

#### Standardized Length Measurements

The body length of the participant was measured twice by investigators to the nearest 0.1 cm using the standardized WHO technique [23]. As the participants were younger than 2 years old, measurements were taken supine by 2 investigators using an infant length board. The average of the 2 measurements was recorded as the participant’s body length and used as input to the LAI algorithm. If the 2 measurements differed by more than 0.5 cm, a third measurement was taken and the average of the three measurements was used.

#### User Experience Questionnaires

Customized questionnaires were used to capture user feedback from investigators and parents on their experience with taking suitable photos according to the study requirements and on other items relating to using a digital measurement tool, including expected accuracy and desirable features.

### LAI Algorithm Overview

**Figure 1:**
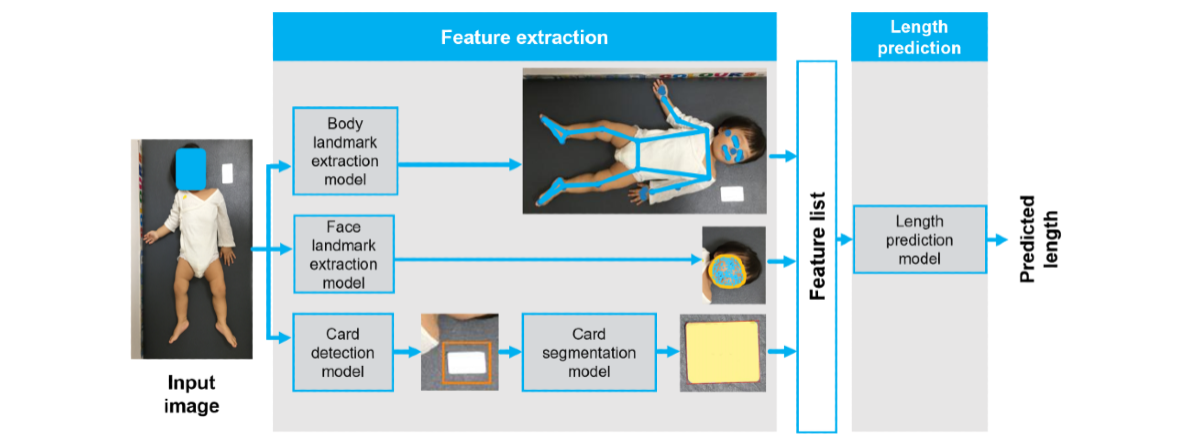
LAI algorithm overview. From an input image of a child in a supine position and a standard reference object, anthropomorphic landmarks of the body and face are extracted, along with the detection and segmentation of the reference object (a standard size card, 85.6 mm by 54.0 mm). These are used by the LAI algorithm to predict the length of the child. LAI: length artificial intelligence.

The LAI algorithm uses state-of-the-art imaging and machine learning techniques to estimate a participant’s length from a single image, such as a smartphone photo. The current algorithm was designed to predict the length of children up to 18 months. The input to the algorithm is a digital image of the participant in a supine position and a reference object (standard size card, 85.6 mm by 54.0 mm). The first step involves extracting image features for both the participant and the reference card (Figure 1). Landmark extraction models are used to detect landmarks on the participant’s face and body (shoulders, hips, knees, ankles, heels, etc) within the image. These estimate the length of individual body segments in pixels. The card detection and card segmentation models are used to locate the reference card in the image and compare its pixel dimensions against its known physical dimensions to generate a pixel per metric value. The feature extraction step thus generates a set of quantitative features used in the length prediction step (Figure 1). A model incorporating these features predicts the total body length in millimeters. The algorithm returns a predicted length value as output only if the key feature extraction steps (body and card features) are successful.

### Image Datasets

The investigator and parent datasets consisted of all images taken by the investigators and the parents, respectively.

### Image Requirements

**Figure 2:**
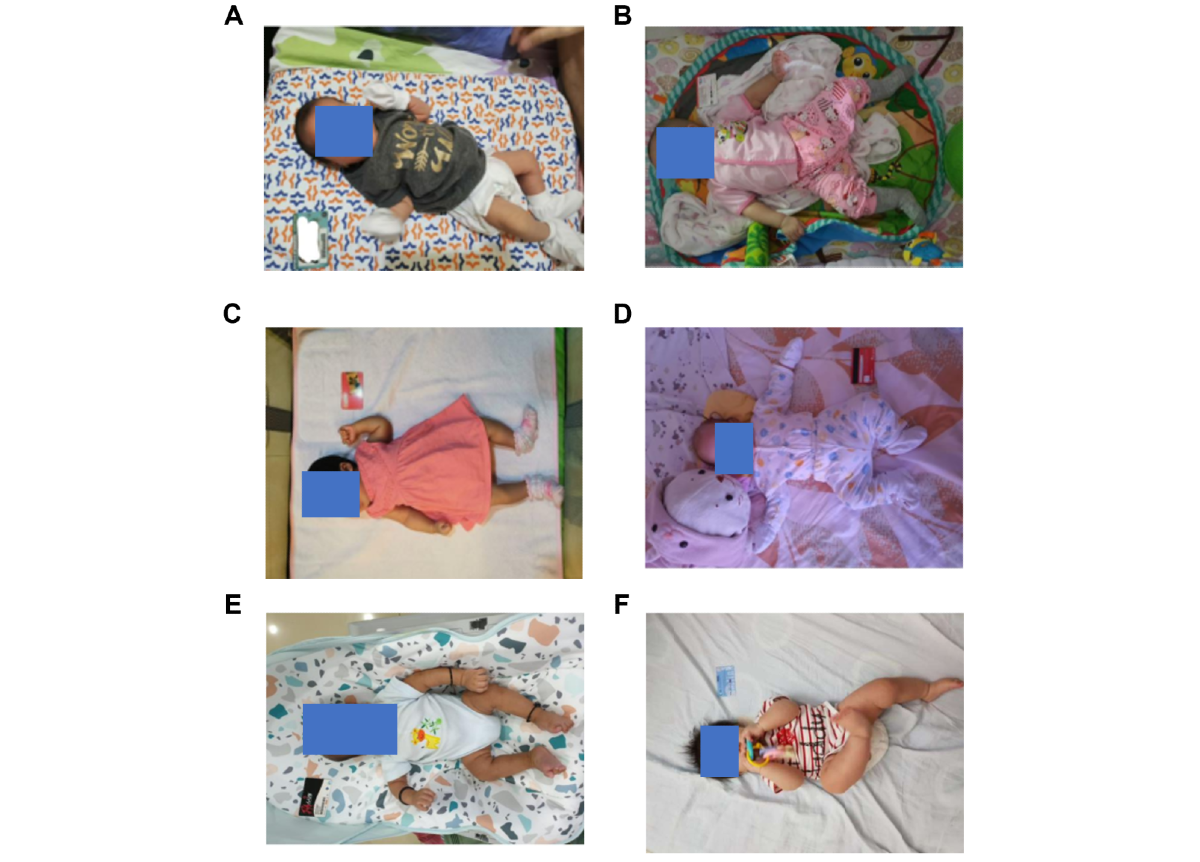
Challenges with image-based length prediction. Images that pose challenges for length prediction by the LAI algorithm include those where (A) the camera is not positioned perpendicularly (90-degree angle) above the participant during image capture, (B) the participant and reference card are not placed on a flat horizontal surface, (C) there is blurring or glare, (D) there are baggy clothes on participant affecting the visibility of body contour, (E) there is low contrast of the participant with background, and (F) the face or body is not fully visible. LAI: length artificial intelligence.

To maximize the number of images usable for length prediction, photo-taking guidelines were given to investigators and parents. This included no clothing on the head or feet (eg, cap, socks, etc); no loose or baggy clothing to ensure that the body contour was visible; high contrast between the background, participant, and reference card; participant and reference card placed on the same flat and stable surface; participant positioned not more than 10 cm from the reference card; taking the image at an angle of 90 degrees from the surface on which the participant and card were placed; and participant’s legs not bent with the entire body visible to the camera. Images that fail to meet these requirements, such as those shown in Figure 2A-F, may fail to generate length predictions.

**Figure 3:**
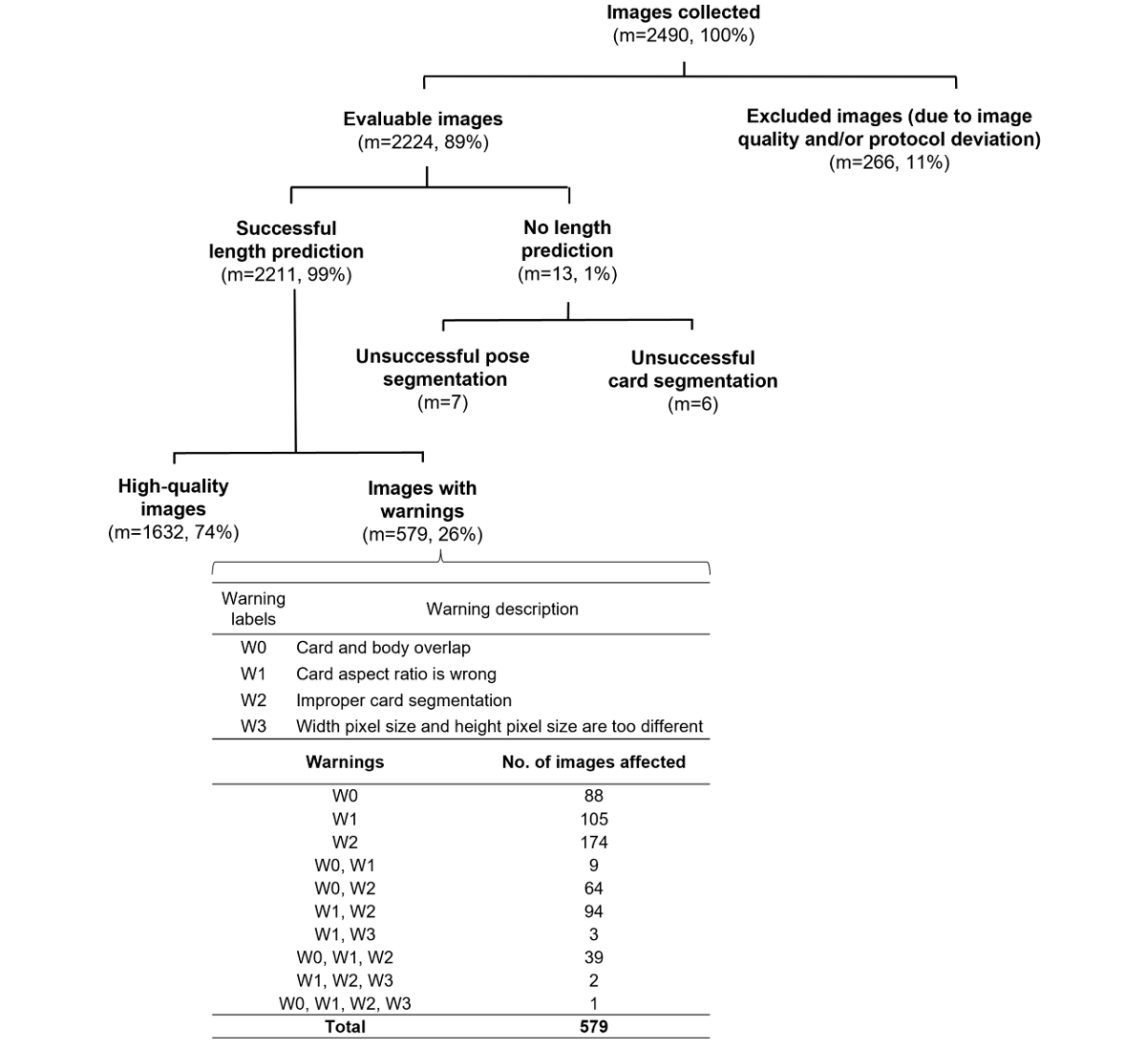
Schematic diagram of image flow. A total of 2490 images were collected in this study and 2224 images were analyzed. A total of 266 images were not analyzed due to protocol deviations (resubmission of images, submission of images outside the stipulated visit window, or images that did not meet the requirements). Of 2224 images analyzed, 2211 images produced a length prediction. The algorithm did not produce a prediction for 13 images due to unsuccessful pose segmentation (m=7) or unsuccessful card segmentation (m=6). High-quality images refer to images that did not generate any warnings. m (%): number and percentage of images in the specified category.

Following further testing and optimization, automated flags (warnings) were incorporated into the LAI (Figure 3). This allows the tool to detect uploaded images that do not meet the specified requirements and warn users that length prediction may be unsuccessful. In total, 4 different types of warnings were implemented.

### Model Training and Performance Metrics

The LAI model was trained on the set of images collected by investigators (investigator dataset) and the investigator-generated length measurements. As described above, a set of features was generated, along with warnings. Only images that did not produce warnings were used to train the model. A 5-fold cross-validation was performed. Cross-validation is a statistical resampling method commonly used in applied machine learning to evaluate model performance with limited datasets [24,25]. The dataset was divided into 5 folds (subsets of data used for model training and testing) using 3 criteria: each participant’s images should appear in only 1-fold; similar numbers of participants and images per fold; and similar distribution of measured body lengths across folds. In addition, details are provided in the *Cross-Validation Procedure* section in Multimedia Appendix 1. A bagged model was used for prediction. Hyperparameter optimization (HPO) incorporated a random feature selection step and was implemented using a state-of-the-art HPO framework [26]. The training was performed using a 2-step process. In the first step, a model was trained on all images from the investigators in the current training fold. A subset of these images within the 90th percentile of the training error was then selected, and the model was retrained on these images to ensure that outliers did not affect the training. Finally, all test images (from the investigators and parents) without warnings within the current fold were used to predict and calculate validation errors. The average validation error from all folds was used to drive the HPO framework to find the best model.

Performance metrics were calculated on a per-image and per-participant basis. For a given image *i*, the body length (*p_i_*) predicted by the trained model was compared with the corresponding participant’s WHO-standardized length measurement (*m_i_*) to derive the following performance metrics: error (*E_i_*, cm: difference between measured and predicted length *E_i_* = [*m_i_ – p_i_*]); absolute error (*AE_i_*, cm: absolute value of the error *AE_i_* = *E_i_*); and absolute percentage error (*APE_i_*, %: AE as a percentage of the length measurement, 
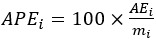
). Bias (average of *E*, cm; 
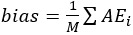
), mean AE (MAE; average of AE, cm), mean APE (average of APE, %), percentages at each AE cutoff (≤1 cm, ≤2 cm, ≤5 cm, and ≤10 cm), and percentages at each APE cutoff (≤2%, ≤5%, ≤10%, and ≤20%) were also calculated. Missing values due to errors, where the model did not return a predicted length value, were counted and reported separately. For participants with successful length predictions based on at least 9 images, the predictions for each image were averaged to generate a single predicted length for that participant.

The performance of the LAI model was evaluated using 5-fold cross-validation performed on the combined investigator + parent datasets and investigator-generated length measurements. To assess the LAI algorithm’s performance relative to measurements in general clinic settings [8,22], the appropriate performance metrics were compared with published values for the technical error of measurement (TEM), an index commonly used in anthropometry to assess the accuracy and reliability of measurements [19-21].

### Statistical Analysis

Due to the exploratory nature of this study, there was no formal sample size calculation. It was estimated that a complete dataset from 200 participants (standardized length measurements, images taken by investigators, images taken by parents, and completed questionnaires from investigators and parents) would allow model performance to be adequately assessed. Assuming a 20% dropout rate, 250 participants were planned for enrolment.

Descriptive statistics were used to summarize participant characteristics and user experience questionnaire responses. Continuous variables were summarized using mean, median, and minimum and maximum values. Discrete variables were summarized using percentages and frequencies by category, including missing values. No statistical testing of formal hypotheses was conducted.

## Results

### Characteristics of Participants and Image Data

In total, 215 participants were enrolled in the study, of whom 50.7% (n=109) were female. The mean age was 6.1 (SD 5; range: 0.0-17.7) months and the median age was 4.4 months. All participants completed the clinic-based data collection procedures, and 200 participants completed both clinic-based and home-based data collection procedures to provide a complete dataset of length measurements, images, and questionnaires.

A total of 2490 images were taken and uploaded (1290 images by 8 investigators at the clinic and 1200 images by 200 parents at home; Figure 3). Of these, 89.3% (2224/2490) of images were analyzed, and 10.7% (266/2490) were excluded due to image quality or protocol deviations.

### Length Prediction Performance of the LAI Algorithm

**Figure 4:**
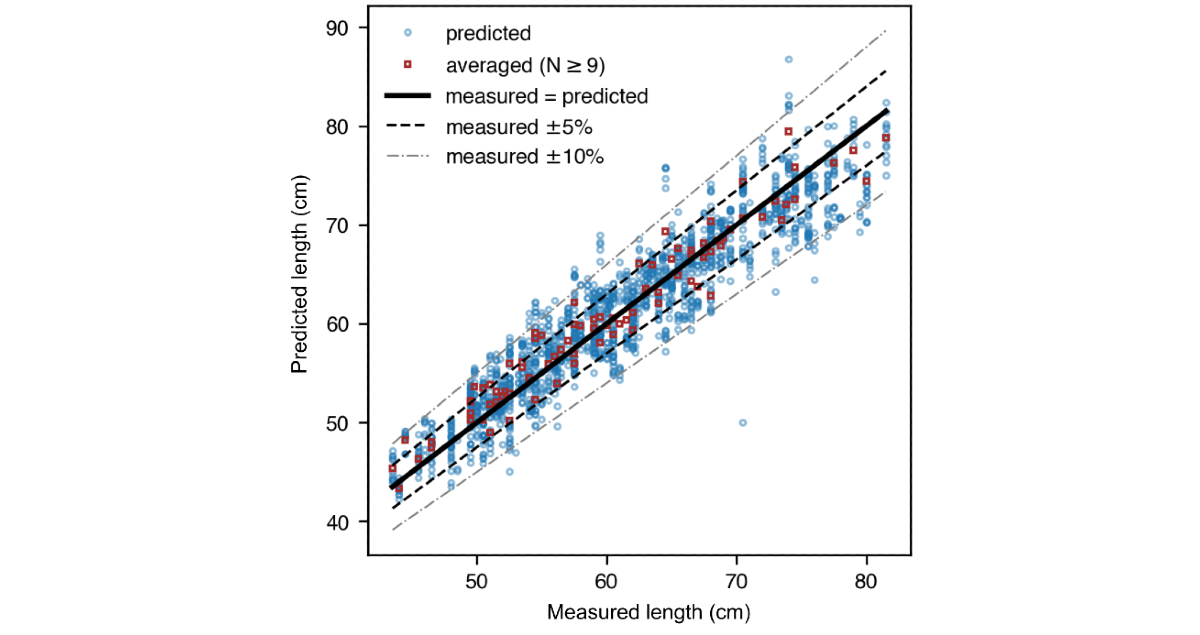
Scatter plot depicting length predictions made by the model versus gold-standard length measurements made by the investigators. For length predictions on individual images, the majority fell within 10% of the participant’s measured length. For averaged length predictions (per participant, for participants who had predictions from ≥9 images), the majority fell within 5% of the measured length. Blue circles represent predictions from all individual images. Red squares represent averaged predictions for children who had predictions from at least 10 images. The thick black line indicates the ideal prediction (ie, length prediction equal to the measured length). Dashed lines represent 5% and 10% deviations from the ideal prediction.

The LAI produced a length prediction for 2211 (99.4%) out of 2224 images (Figure 3). In total, 0.6% (13/2224) of images did not produce a prediction due to either unsuccessful pose segmentation (7 images) or unsuccessful card segmentation (6 images). For the set of 1632 high-quality images (those that did not generate any warnings; Figure 3), the bias (mean error) for individual image predictions was minimal (0.03 cm; Table S1 in Multimedia Appendix 1). Most of the length predictions for these individual images (1557/1632, 95.4%) were within 10% of the measured length (Figure 4 and Table S1 in Multimedia Appendix 1). We found that length prediction was improved by averaging over multiple images for a participant. For 88 participants who had predictions for ≥9 images, the majority of these averaged length predictions (71/88, 81%) were within 5% of the measured length (Figure 4 and Table S1 in Multimedia Appendix 1).

**Figure 5:**
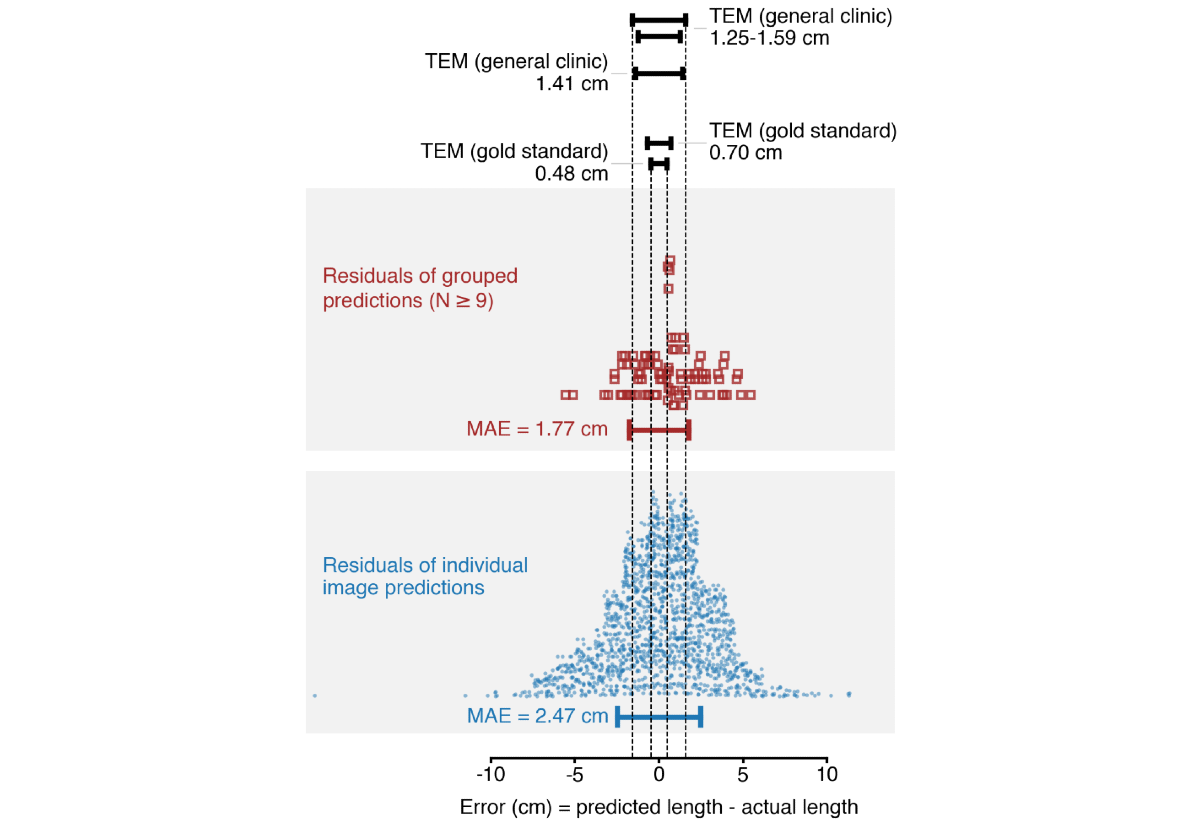
The overall distribution of errors (residuals) for individual image predictions (blue dots) and participant-averaged predictions made by the model. These were presented alongside published interobserver TEMs of “gold standard” length measurements from WHO (0.48 cm and 0.70 cm) and general clinics or community health settings (1.41 cm and 1.25-1.59 cm). The MAE of individual image predictions was 2.47 cm. When averaged, the predictions had an MAE of 1.77 cm, which approaches the TEM range reported in general clinics. MAE: mean absolute error; TEM: technical error of measurement; WHO: World Health Organization.

The overall distributions of errors for individual image predictions and participant-averaged predictions are illustrated in Figure 5 [8,19,20,22]. Published interobserver TEM ranges for length measurements from the WHO Multicenter Growth Reference Study (0.48 cm [19], 0.70 cm [20]) and general clinics or community health settings (1.41 cm [22], 1.25-1.59 cm [8]) are indicated on the figure for comparison. The MAE for individual image predictions was 2.47 cm, and the MAE for participant-averaged predictions was 1.77 cm, which approaches the TEM range reported in general clinics or community health settings [8,22].

**Figure 6:**
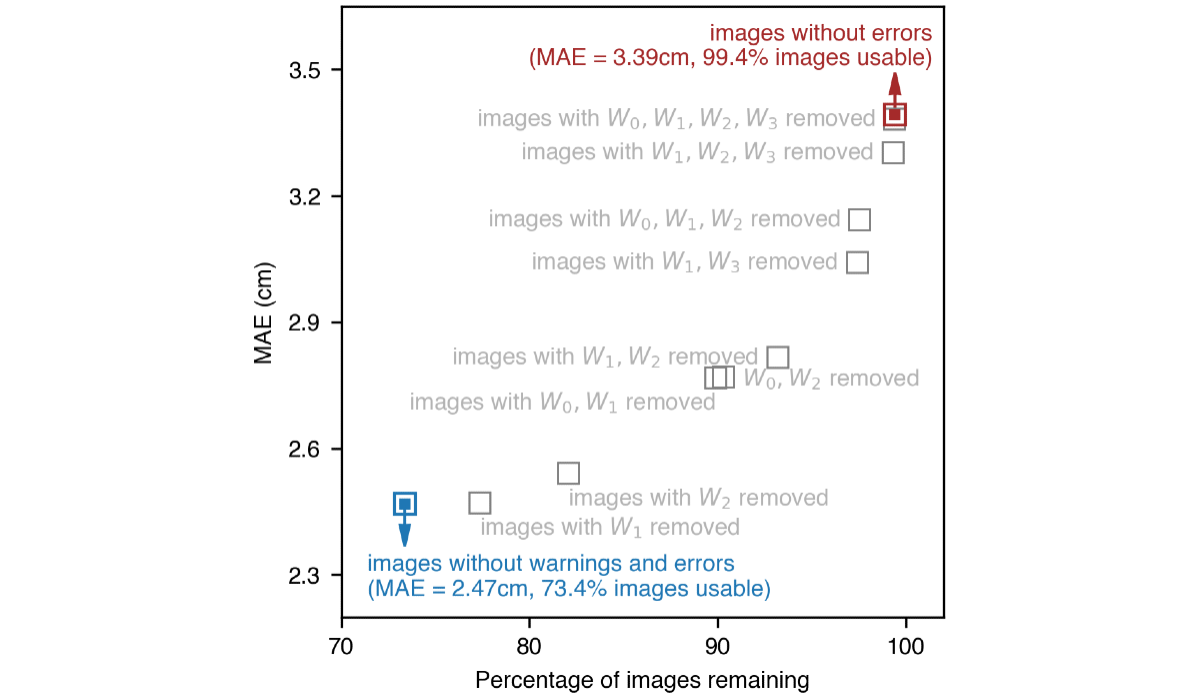
A plot illustrating the percentage of images available for length prediction and the corresponding MAE under varying scenarios where combinations of different warnings were ignored. Ignoring more warning types allowed more images to be used but yielded less accurate length predictions. Warnings: W0, card and body overlapping; W1, incorrect card aspect ratio; W2, improper card segmentation; W3, width, and height pixel size are too different. MAE: mean absolute error.

A quarter of the images with successful length predictions (579/2211, 26.2%) generated at least 1 warning **(**Figure 3). For this study dataset, the most common warning was improper card segmentation. The numbers of images affected by each type of warning are shown in Figure 3. Figure 6 illustrates the percentages of images available for length prediction under scenarios where different warning types are ignored, and the corresponding MAE values. The length prediction workflow can be adjusted to use more or less stringent settings, which affects the number of images retained for prediction and prediction error. Retaining fewer but higher quality images (fewer warnings) for prediction resulted in smaller MAE values (2.47 cm on images without any warnings); conversely, ignoring more warning types allowed more images to be used, but led to an increased MAE (3.39 cm using all images regardless of warnings). Similarly, the MAE for participant-averaged predictions decreased from 2.48 cm (n=155) to 1.77 cm (n=88) when only high-quality images without warnings were used for prediction (Table S1 in Multimedia Appendix 1).

### User Experience

**Figure 7:**
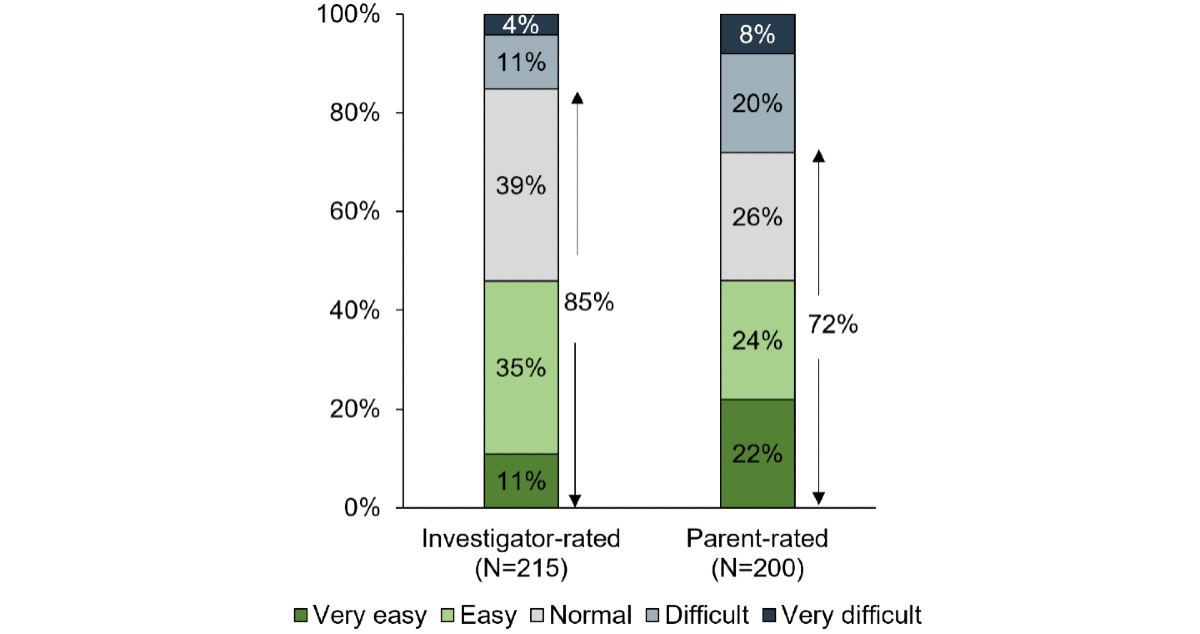
Ease of collecting images as rated by investigators and by parents. N represents the number of participants for which the investigator or parent provided a rating.

In most cases, investigators and parents reported that they did not find it difficult to capture the required images (Figure 7). Investigators and parents rated the photo-taking process as very easy, easy, or normal for most participants (182/215, 84.7% participants, and 144/200, 72% participants, respectively).

#### User Feedback on the Use of a Digital Tool for Length Measurement

In total, 7 (88%) of 8 investigators indicated that they would be likely or very likely to use a digital tool that could automatically measure a child’s length from an image, if available for clinical use (Multimedia Appendix 2). A similar proportion (7/8, 88% investigators) reported that they would be likely or very likely to recommend a digital length measurement tool to parents for home use, if available.

As for parents, most (143/196, 73% responses) were not currently taking length measurements regularly (at least once a month or more frequently) at home, whereas 57.1% (112/196) never measured length at home and 16.3% (32/196) measured less frequently than once a month (Multimedia Appendix 3). However, 91.5% (183/200) felt that a digital tool that could automatically predict length from an image would be useful or very useful for them to measure their child’s length at home (Multimedia Appendix 3), and 88.5% (177/200) indicated that they would use such a tool at least once a month or more frequently (Multimedia Appendix 3).

Investigators and parents were asked about the magnitude of difference between standard clinic measurements and the length predicted by a digital tool that they would find acceptable in their typical use settings. For all investigators, only differences of ≤1 cm (4/8, 50% investigators) or ≤2 cm (4/8, 50% investigators) with respect to clinic measurements were considered acceptable (Multimedia Appendix 4). For most parents, differences of ≤1 cm (63/200, 31.5%) or ≤2 cm (93/200, 46.5%) were deemed acceptable, although a small proportion of parents considered differences of up to 5 cm acceptable.

#### Additional Desirable Features for an Automated Growth Measurement Tool

Apart from automated length measurement, investigators and parents highlighted several additional features that would be desirable in an image-based tool. Key desired features included estimating other anthropometric measurements, such as weight, head circumference, and growth-tracking functionality. Half of the investigators (4/8, 50%) suggested it would be useful to estimate other anthropometric parameters. A quarter (2/8, 25%) of investigators indicated that having a growth-tracking function would help detect growth abnormalities. In addition, 16% (32/200) of parents wanted the tool to be able to estimate weight or other anthropometric measurements. In total, 40% (80/200) of the parents wanted to be able to track their child’s height, length, or both over time and have these measurements presented alongside the corresponding WHO height-for age and length-for-age charts.

## Discussion

### Principal Findings

Length measurement is challenging in clinical practice, especially among children younger than 2 years of age, where inaccuracy and interobserver variability in length measurements have been documented in primary care and community health settings [5,8,27]. Without proper equipment and training, it is impractical to expect parents and caregivers to take accurate and reliable length measurements at home regularly. Approaches for computer-assisted body length estimation from digital images have been proposed but involve elaborate requirements for data capture or procedures that may cause disturbances to the child [17,18] and limit their real-world feasibility and applicability. In contrast, the LAI algorithm was designed to have minimal requirements in terms of equipment and user training: only a smartphone and a readily available reference object (standard-size credit card) are required for data collection.

In our study, both parents and investigators found it easy to take the required photos for automated length estimation. Results from this proof-of-concept study suggest that the LAI algorithm, when trained on high-quality length measurements and image data, can predict children’s body length with minimal systematic error and accuracy approaching that achieved in general clinics or community health settings. This indicates the potential for future implementations of the LAI algorithm to make growth monitoring more accessible to nonexpert users. The MAE for individual image predictions was 2.47 cm, and our results suggested that length prediction could be improved by averaging across multiple images of a child: participant-averaged predictions had a smaller MAE of 1.77 cm, which approaches the published interobserver TEM range (1.25-1.59 cm) reported in general clinics and community health settings [8,22]. Thus, the LAI algorithm would enable parents to estimate their child’s length using images captured using their smartphones, with accuracy comparable to length measurements in general clinics or community health settings. Published reports indicate that the level of accuracy considered acceptable ranges from 0.5 cm to 2.0 cm, depending on the clinical indication for which length measurements are required [5,6,9,19-22,27]. For clinical growth monitoring, the WHO Multicenter Growth Reference Study protocols recommend interobserver differences of <0.7 cm for length [20], and the Standardized Monitoring and Assessment of Relief and Transitions (SMART) manual indicates an acceptable limit of <0.5 cm for height [28]. Growth measurement studies commonly report differences of ≤0.5 cm for expert anthropometrists [19-21,29]. This is consistent with the views of the study investigators, who indicated that they considered an accuracy of within 1 cm acceptable in an expert clinical setting.

Although smartphones greatly facilitate data collection for LAI algorithm, it is not always possible to consistently capture high-quality images of a child, which affects the accuracy of predictions. Commonly encountered image quality issues include the visibility of body landmarks obscured by clothing or limb position (Figure 2). Within the current LAI algorithm, we implemented a system that detects issues with uploaded images and generates warnings that these could affect length prediction (Table S1 in Multimedia Appendix 1). The settings can be tuned to ignore warnings and allow more images to be used for length prediction but at the cost of generating less accurate predictions. We envision that future versions of the tool will be integrated into a smartphone application that offers real-time feedback to guide parents or caregivers in positioning the baby and the standard-size reference object. Such feedback, including alerts about potential image quality issues and guidance on how to avoid or reduce key sources of error, would help users to take photos that are more likely to result in accurate predictions.

Other technologies, such as depth-of-field sensors available on some consumer devices, could potentially be integrated to further improve performance. Time-of-flight or light detection and ranging sensors can provide depth-of-field information that could be used with the current image-based algorithm to increase length prediction accuracy. Relevant practical considerations include the availability and accuracy of these technologies, especially for smaller participants like infants.

This proof-of-concept study collected images and corresponding length measurements and used these to explore the feasibility of a digital tool that can be used in home environments and in clinics to monitor growth over time. Due to the limited data available, a cross-validation approach was adopted to maximize the information that we could obtain about the tool’s performance. This lack of an external validation sample may introduce limitations such as underestimation of error when the model is applied to new datasets, and systematic errors if the model’s assumptions do not hold on to other datasets. Although care was taken to ensure that the reference measurements were as accurate as possible by using a standardized protocol (WHO method) and trained personnel, we have not formally accounted for the possibility of human error in the length measurements used for training.

Findings from our study have been used to refine the tool further, and projects to evaluate the feasibility and acceptability of the improved version are ongoing. At present, the proof-of-concept results suggest that the LAI algorithm’s current performance may be compatible with personal use, such as general growth tracking at home, as its performance approaches that of manual length measurement in general clinics or community health settings [8,22]. Using the LAI algorithm, parents could record length measurements more frequently and conveniently at home. This idea is consistent with feedback from parents of the children in our study. Most parents reported that they did not measure their child’s length at home; on the other hand, they indicated that an automated measurement tool was desirable and would be used at least monthly or more frequently. Besides automated length measurement, parents wanted a tool capable of estimating other anthropometric parameters and allowing them to track their child’s growth with reference to WHO growth charts [3]. This user feedback provides valuable insights that can guide future development of the tool.

### Conclusion

One of the main strengths of the current LAI approach is its simplicity and practicality for nonexpert end users. Data collection requires no specialized equipment or training; physical discomfort and disturbance to the child are minimized. This innovative approach explored the feasibility of image-based automated body length estimation that can be conveniently performed in a wide range of environments by any user. The potential value of such tools to nonexpert users is underscored by the range of studies that explore the possibilities of digital technology-assisted anthropometry [30,31]. The feedback collected from parents and clinicians in this study will inform future versions of the tool to better cater to the unique requirements of different users. It should be noted that the LAI algorithm’s performance was evaluated only using data from healthy children (those without known growth-related conditions). Further studies with different populations will be needed to guide the design and optimization of the LAI algorithm for use in more specialized clinically oriented tasks such as monitoring for abnormal growth.

It should be noted that this was a proof-of-concept study to demonstrate the feasibility and acceptability of the LAI tool for estimating body length in young children (aged <18 months) in the home and similar environments. The performance achieved to date suggests that the current version of the LAI algorithm would not replace the standard clinical method used by health care professionals, but there is potential for future development to enhance its accuracy and applicability. Although the tool does not currently meet the requirements for highly accurate measurement in specialist clinical applications, our findings suggest that improving image quality is one way to increase length prediction accuracy. Integration of other technologies, such as depth-of-field sensors available on some consumer devices, could be explored to further improve performance. The current performance of the LAI algorithm, coupled with its ease of use, suggests it has the potential to be a feasible method of measuring a child’s length for use by parents with accuracy approaching that of general clinic or community health settings.

## Appendix

Multimedia Appendix 1 Cross-validation procedure, important limitations of the current LAI method and potential solutions, and performance of LAI for length prediction.

Multimedia Appendix 2 Investigator ratings (N=8) on how likely they were to use and recommend a digital tool to measure a child’s length in a clinical setting. N represents the number of respondents.

Multimedia Appendix 3 Parents’ (N=200) assessment of the (A) frequency of length measurement in the home in terms of how often they currently measure their child’s length manually at home and how often they would measure their child’s length if they had a digital tool that could automatically measure length from an image, and (B) usefulness of such a digital tool. N represents the number of respondents.

Multimedia Appendix 4 Acceptable magnitude of difference between LAI-predicted length and standardized length measurement for investigators n=8) and parents (n=200). N represents the number of respondents. LAI: length artificial intelligence.

## Abbreviations

AE: absolute error
APE: absolute percentage error
HPO: hyperparameter optimization
LAI: length artificial intelligence
MAE: mean absolute error
SMART: Standardized Monitoring and Assessment of Relief and Transitions
TEM: technical error of measurement
WHO: World Health Organization
