# Supplementary material for: Exploring the Use of a Length AI Algorithm to Estimate Children’s Length from Smartphone Images in a Real-World Setting: Algorithm Development and Usability Study
Source: JMIR Pediatr Parent. 2024 Nov 22;7:e59564. doi: 10.2196/59564 (PMC11624450; doi:10.2196/59564)
Supplement: Multimedia Appendix 1 [file pediatrics_v7i1e59564_app1.docx]

# **Multimedia Appendix 1: Supplementary Material**

## Cross-validation procedure

Five-fold cross-validation was performed. Folds were constructed to ensured that the train and test sets did not contain images from the same infants, and that there was a roughly equal distribution of the measured lengths in each fold. The process is as follows:

First, all images with warnings were excluded from the folds. The remaining images were assigned to the folds based on subject IDs, keeping the total number of subjects in each fold approximately equal. The corresponding Investigator and Parent images were based upon the available unique subjects assigned to each fold.

| **Fold  number** | **Unique subjects** | **Investigator images** | **Parent  images** | **Total  images** |
| --- | --- | --- | --- | --- |
| 0 | 42 | 220 | 117 | 337 |
| 1 | 42 | 202 | 148 | 350 |
| 2 | 42 | 222 | 113 | 335 |
| 3 | 42 | 220 | 111 | 331 |
| 4 | 39 | 183 | 96 | 279 |

An additional condition was imposed to achieve a roughly equal distribution of measured lengths across the 5 folds, as shown in the figure below. Note that this can only be approximately equal due to the other conditions described above (unique subjects in each fold, roughly equal numbers of subjects and images per fold).


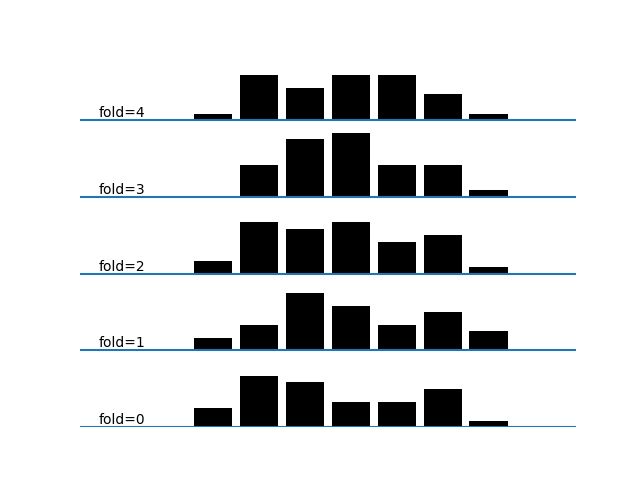


Finally, the cross-validation process proceeded as follows:

For the first fold, all images in fold 0 (220 Investigator + 117 Parent images) were used as validation images, and Investigator images from the other folds (=202+222+220+183) were used for training. A similar approach was followed for the other 4 folds. After this process was completed for all 5 folds, the predictions for all the validation images were consolidated to generate the overall prediction results.

## Important limitations of the current LAI method and potential solutions

Limitations related to the photo-taking setup

1. **Problem 1**: One key assumption of the method is that the camera angle is perfectly perpendicular to the plane of the child’s body (the flat surface on which they are lying). If pictures are taken at other angles (greater or less than 90 degrees), different parts of the body will be closer/further from the camera, and this will lead to an inaccurate result.
   **Potential improvement**: It might be possible to use the gyro sensor available in most smartphones to help correct for this. For example, this could be implemented by using the gyroscope sensor to ensure that the user is only able to take a picture when the camera is pointing vertically down. However, this assumes that the surface on which the child lies is also horizontal.
2. **Problem 2**: Another assumption is that the camera is sufficiently far away from the child that the rays of light reflected from the child can be considered parallel. This is not always the case. Body parts closer to the camera will appear to be larger than those further away.
   **Potential improvement**: It might be possible to correct for this issue by using the depth sensor available in many smartphones (such as LiDAR or ToF sensors) to ensure that images are taken at a suitable distance from the child.
3. **Problem 3**: The model has difficulty identifying the “correct” reference object, resulting in unsuccessful card segmentation. This is currently one major source of error, as the reference object segmentation step is not coded to use specific visual features to recognize the correct credit card-sized rectangular object within the image.
   **Potential Solutions**: One possibility is to use a unique reference object bearing a QR code or distinct visual feature that users can print out and place next to the child. Additionally, the user interface could be designed to allow the user to visually confirm the reference object (e.g., displaying a bounding box outline) and point to it, if necessary, through a touch interface. However, this would introduce additional steps for the user (e.g., printing out the reference object), which would make the process less convenient.

Limitations related to the algorithm or measurement processes

1. **Problem:** Inaccuracies of sub-models. As the LAI model is based upon a number of other sub-models (such as the pose landmark model, the reference object detection model, and the reference object segmentation model), inaccuracies of these models will contribute to the inaccuracy of the overall model.
   **Potential Solution**: Models for standard vision tasks such as object detection, segmentation and pose estimation continue to improve in accuracy, speed and memory footprint over time. New model architectures will be used to improve model accuracy as they emerge. Further improvement could be achieved over time through machine learning pipelines trained with larger quantities of labelled data.
2. **Problem:** Model accuracy has been determined with respect to investigator-measured body length using the WHO method. Under this evaluation scheme, the human-derived measurements used for training are assumed to be entirely accurate, which may not be the case. Hence, part of the MAE that we report may include measurement errors in the training data.
   **Potential Solution**: Evaluation methods that can potentially distinguish measurement error from the error of the model prediction should be developed so that the true error of the AI’s prediction that does not include human error may be calculated, allowing the real value of the model quality to be identified.
3. **Problem**: Fundamental limitations of human pose detection – ultimately, defining body landmarks are an inexact science (for example, the exact location of a hip). Joints in the human body are bigger than several millimeters, and thus millimeter-level precision in estimating the location of a hip or knee is not a very well-defined problem. Furthermore, unlike the manual measurement method, there is no requirement for the infant to fully stretch out. With infants that are not stretched out, centimeter-length differences may therefore be expected.
   **Potential Solution**: Addressing all of these limitations would require fundamental changes to the design assumptions of the algorithm/process that are beyond the scope of this proof-of-concept study.

## Table S1. Performance of LAI for length prediction

| **Performance metrics** | **Images with warnings** | | **Images without warnings** | |
| --- | --- | --- | --- | --- |
|  | **Individual image predictions**  **(*m*=2211)** | **Averaged predictions^1^**  **(M=155)** | **Individual image predictions**  **(*m*=1632)** | **Averaged predictions^1^**  **(M=88)** |
| Error (E) |  |  |  |  |
| Bias (mean error), cm | 0.20 | 0.50 | 0.03 | 0.49 |
| Absolute error (AE) |  |  |  |  |
| Mean, cm | 3.39 | 2.48 | 2.47 | 1.77 |
| ≤1 cm, *m* (%) | 556 (25%) | 52 (34%) | 426 (26%) | 35 (40%) |
| ≤2 cm, *m* (%) | 1049 (47%) | 90 (58%) | 826 (51%) | 56 (64%) |
| ≤5 cm, *m* (%) | 1834 (83%) | 137 (88%) | 1446 (89%) | 85 (97%) |
| ≤10 cm, *m* (%) | 2123 (96%) | 151 (97%) | 1624 (100%) | 88 (100%) |
| Absolute percentage error (APE) |  |  |  |  |
| Mean, % | 5.44 | 4.06 | 4.04 | 2.99 |
| ≤2%, *m* (%) | 658 (30%) | 63 (41%) | 505 (31%) | 41 (47%) |
| ≤5%, *m* (%) | 1412 (64%) | 115 (74%) | 1121 (69%) | 71 (81%) |
| ≤10%, *m* (%) | 1988 (90%) | 148 (95%) | 1557 (95%) | 88 (100%) |
| ≤20%, *m* (%) | 2158 (98%) | 153 (99%) | 1631 (100%) | 88 (100%) |

^1^For children who had length predictions from at least nine images, the predictions were averaged

*m*, number of images that yielded a length prediction; M, number of subjects with averaged length predictions

LAI, Length Artificial Intelligence
