# Supplementary figures and images for: Exploring the Use of a Length AI Algorithm to Estimate Children’s Length from Smartphone Images in a Real-World Setting: Algorithm Development and Usability Study
Source: JMIR Pediatr Parent. 2024 Nov 22;7:e59564. doi: 10.2196/59564 (PMC11624450; doi:10.2196/59564)

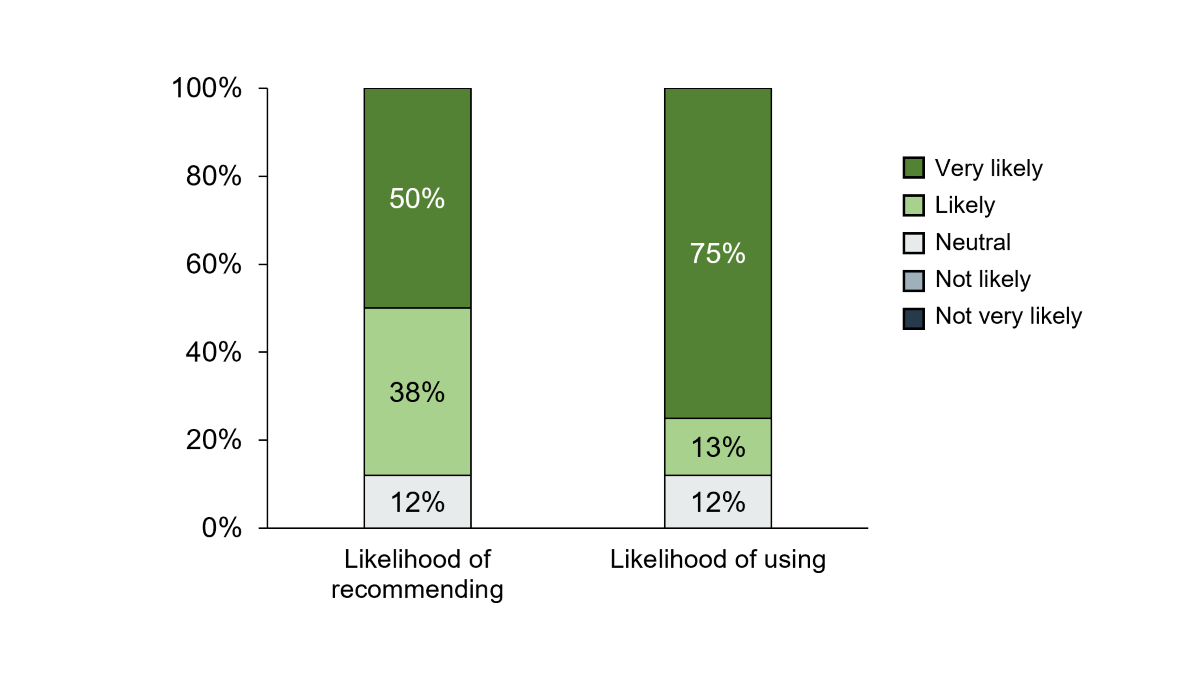

Supplement: Multimedia Appendix 2 [file pediatrics_v7i1e59564_app2.png]

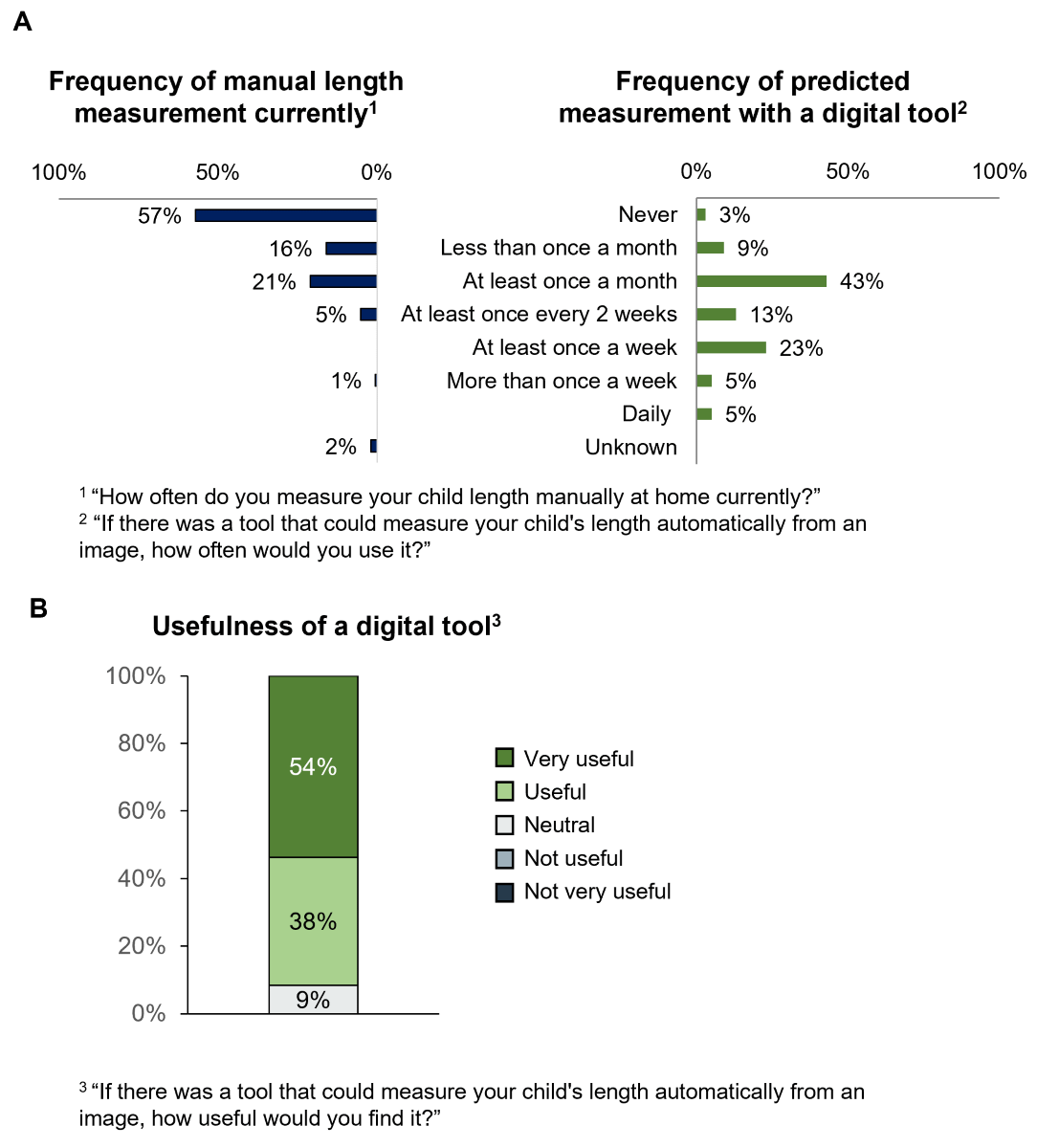

Supplement: Multimedia Appendix 3 [file pediatrics_v7i1e59564_app3.png]

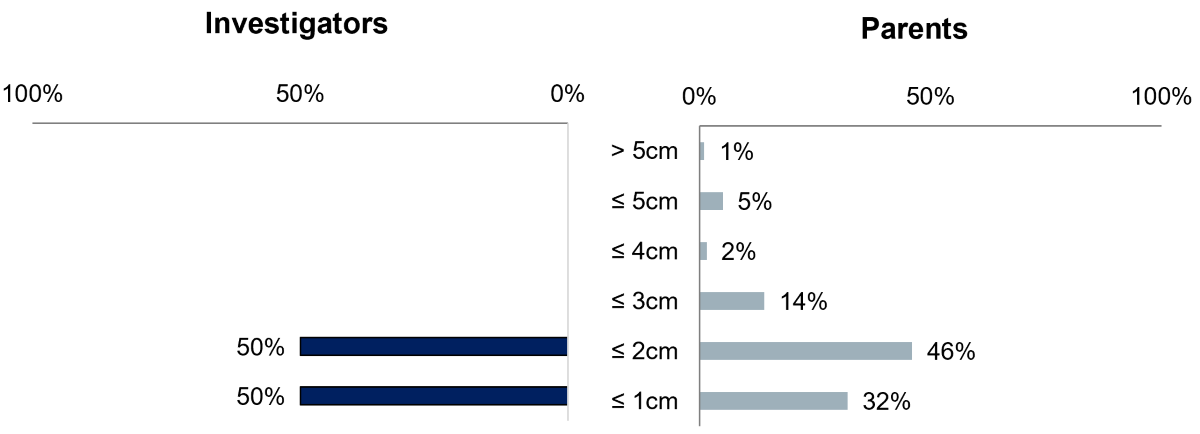

Supplement: Multimedia Appendix 4 [file pediatrics_v7i1e59564_app4.png]
